# The power of symbolic capital in patient and public involvement in health research

**DOI:** 10.1111/hex.12519

**Published:** 2016-11-24

**Authors:** Louise Locock, Anne‐Marie Boylan, Rosamund Snow, Sophie Staniszewska

**Affiliations:** ^1^ Health Experiences Research Group Nuffield Department of Primary Care Health Sciences University of Oxford Oxford UK; ^2^ Oxford NIHR Biomedical Research Centre Oxford UK; ^3^ WMS—Royal College of Nursing Research Institute University of Warwick Coventry UK

**Keywords:** Bourdieu, patient and public involvement, power, qualitative research

## Abstract

**Background:**

Policy‐makers and health research funders increasingly require researchers to demonstrate that they have involved patients in the design and conduct of research. However, the extent to which patients and public have the power to get involved on an equal footing is dependent on their economic, cultural, social and symbolic capital.

**Objective:**

To explore power relations in patient and public involvement (PPI) in research, particularly how patients may wield symbolic capital to develop a more equal relationship.

**Methods:**

Narrative interviews with a maximum variation sample of 38 people involved as patients, carers or public in health research, analysed thematically.

**Findings:**

Symbolic capital may be demonstrated in a range of ways (sometimes alongside or in the absence of other forms of capital): illness experience, technical illness knowledge and the challenging outsider. Symbolic capital is unstable and dependent on others for recognition and legitimacy. Nonetheless, participants identify a gradual shift in power relations over time.

**Discussion and conclusions:**

Research into PPI has been conceptually and theoretically poor, limiting our understanding of its mechanisms and wider contextual elements. Our findings demonstrate the importance of reflecting on the forms of power and capital wielded by the health research community, and of acknowledging the way in which PPI is challenging the status quo. As one of the first papers to conceptualize how different forms of symbolic capital operate and their critical role in challenging the balance of power, our findings may help researchers better plan their PPI activities and reflect on their own power.

## 1 INTRODUCTION—PATIENT AND PUBLIC INVOLVEMENT (PPI) IN RESEARCH

The principle that health care should be designed to be more person‐centred and that individuals have a right to make decisions about treatment and manage their own health, is now firmly established as policy in many health systems—even if practice sometimes lags behind principle.^1^, ^2^ Patient involvement collectively in decisions about health service design and organization has also long been advocated,^3^, ^4^ although again the reality may not match the rhetoric.^5^, ^6^, ^7^

The parallel principle that patients should be closely involved in decisions about health research has evolved over the last two decades.^8^, ^9^ In the UK, the National Institute for Health Research advisory group supporting patient and public involvement (or “PPI”) in health research, NIHR INVOLVE, defines involvement as “research being carried out “with” or “by” members of the public rather than “to,” “about” or “for” them.”^10^

In the UK, it has become common practice for research funders, especially the NHS National Institute for Health Research (NIHR), to require researchers to demonstrate public involvement in their grant applications—or justify why they do not think it appropriate. The British Medical Journal now requires all papers to include a section on PPI. Biomed Central has launched a journal specifically on the evidence base for PPI, *Research Involvement and Engagement*.

### 1.1 Background—PPI and power relations

The Chief Medical Officer for England and Wales, Dame Sally Davies, has stated that “No matter how complicated the research, or how brilliant the researcher, patients and the public always offer unique, invaluable insights. Their advice ….invariably makes studies more effective, more credible and often more cost efficient.”^11^

It is arguable that this statement is more aspirational than evidence based; while a few studies of PPI in research have demonstrated some impacts, the quality of the evidence remains variable.^12^ This is compounded by what has been termed a “cycle of tokenism”^9^: if involvement is undervalued or resisted by researchers and conducted tokenistically, it then fails to show any impact, reinforcing researchers’ original scepticism.

It follows that the relationship between researchers and PPI contributors is a crucial mediator; at the heart of this relationship is the exercise of power. Beresford,^8^ Gibson, Britten and Lynch^13^ suggest involvement cannot be divorced from broader politics and ideology and reduced to a simple “what works” question. They argue for an emancipatory approach, in which patients and professionals participate in research as equals, recognizing the different forms of knowledge and expertize they bring as equally valid rather than devaluing experiential forms of evidence. This reflects the most recent policy review which has identified the core concept of co‐production as underpinning the future development of PPI^14^, potentially blurring professional boundaries and challenging current power relationships.

However, as Gibson, Lewando‐Hundt and Blaxter^15^ point out, the reality of public involvement has rarely come near this ideal. Drawing on Nancy Fraser's^16^ work on “weak” and “strong” publics, they conclude that power is not equally distributed and “participatory parity” is hard to achieve. Most involvement has been more akin to a “weak public” (where people discuss the topic but have little chance to influence real decisions) than a “strong public” (where people can exert real influence or even make decisions).

Hutchison, Rogers and Entwistle^17^ have also argued that in health research, patients should be seen as bringing a unique knowledge as equals, but in practice “disciplinary indicators of credibility in clinical and academic health research contexts might be wrongly applied to those involved in PPI, undermining their potential to contribute” (p1 epub).

Professionals shape and control how people get involved in numerous ways, from selecting whom to involve or exclude and at what stage, privileging or dismissing certain types of knowledge, through to agenda setting or meeting at times or locations which make it hard for some people to attend.^15^, ^18^ These actions may be less or more deliberate, but they underline the fact that professionals hold most of the power, and PPI depends at least in part on how much power professionals are willing to cede. At the same time, this must be seen in the broader context of continuing renegotiation of professional power and hierarchical dominance from the second half of the 20th century onwards.

Stephen Lukes’ three dimensions of power provide a framework for analysing how researcher practice affects involvement.^19^ Lukes defines power thus: “A exercises power over B when A affects B in a manner contrary to B's interests.” This may be achieved through:

1. overt domination;
2. suppressing certain topics (keeping things off the agenda, making it difficult for some things to be said or some voices to be heard);
3. shaping desires—“to get others to have the desires you want them to have—that is, secure their compliance by controlling their thoughts and desires.”

Although Lukes’ ideas have been influential, his analysis focuses mainly on the expression and results of power, but less on the detail of how power is created, maintained or challenged.

Using Bourdieu's work on power and forms of capital (see Box 1) can help understand the processes behind the expression of power and analyse the relationship between PPI advisers^1^ and professionals^13^, ^18^—in this case the research community. The research world is a distinctive habitus, characterized by norms and rules which include the (written and unwritten) rules of the funding application process, the automatic use of formal meetings with agendas and minutes, and the acceptance of a hierarchy of evidence types.

Box 1 **Definition of useful terms from Bourdieu**

1

**Table:** 

Habitus	Habitus is the way one unconsciously acts, interacts and behaves within the social world in a “taken‐for‐granted” manner, according to the socialized norms, traditions and unwritten rules of the particular group you are part of.
Capital	Capital refers to a range of material and symbolic qualities that establish social standing within a particular habitus/social setting. Capital is used to provide increased access to various resources within the field concerned (whether physical, material or symbolic). Power is derived from the configuration of all these types of capital.
Economic capital	Money and material goods—wealth.
Cultural capital	Tastes, values and preferences which may indicate social class and educational background. These tastes establish “distinction” in which the self recognizes others with shared values.
Social capital	Social capital is obtained from a network of relationships with others like yourself, as well as by interacting with those beyond immediate social/peer networks.
Symbolic capital	Perceived levels of status, prestige and respect held by individuals within and beyond immediate social networks.
Field	Field provides context to habitus. Fields are the shared social spaces or arenas that shape and form habitus, frame conduct and provide opportunities for creating capital. An example might be “the education system.” Bourdieu often referred to field as part of a “game” played on a daily basis (famously describing his discipline of sociology as a “combat sport”).
Practice	Autonomous actions and independent decisions that emerge from the interconnected relationships between habitus, capital and field.
Logic of practice	Knowing “how to play the game” and how to successfully negotiate the “field” to one's advantage. This game is played out verbally and physically (on a conscious and unconscious level) on a daily basis (eg at work, at home, in the shopping centre, on public transport).

Bourdieu views academia as “a habitus which disposes agents to retreat to their ivory towers and think and act as if the world were an idea to be contemplated and discussed, rather than a series of problems and issues affecting the everyday lives of people” (p. 19).^20^ PPI seeks to disrupt this habitus by bringing the everyday lives of people into the ivory tower—but this remains inviting people into the researchers’ world rather than meeting on neutral ground.

Some PPI contributors may feel more at home than others in this territory, depending on what kinds of capital they wield. Economic, cultural and social capital^21^ will all be important in understanding power between researchers and patients. For example, there has been debate about whether people who are well‐off, well‐educated and well‐networked are more likely to get involved in public services.^22^ Specifically in research, it has been argued that researchers may involve people who they believe will “understand science,” thus picking people in their own image, such as retired engineers or scientists.^23^ The politics of reimbursing people for PPI and the impact on benefit claimants has also been the focus of much attention.^24^

While other forms of capital have received varying amounts of attention in the involvement and participation literature, in this study we focus especially on symbolic capital as a neglected area. Both Gibson et al^13^ and Callaghan and Wistow^18^ have explored symbolic capital in relation to public involvement in general, and specifically in service planning and management, but the concept has received little attention in research PPI. The possession and display of prestige, status and authority may be a result of (and reinforce) other forms of capital,^25^ but can also be derived from someone's prior experience giving them a particular authority which may appear at odds with a lack of other forms of capital. A common example is returning combat veterans holding special status in society, even though they may have otherwise low capital and face stereotypical judgements.^26^ Illness experience may be another form of symbolic capital which we explore.

In this study, we report findings from a study for NIHR Oxford Biomedical Research Centre of the experiences of people who get involved in research, with a particular focus on what they say about their interactions with researchers, and how they wield symbolic capital to develop a more equal relationship (or not).

## 2 METHODS

Our qualitative study explored the question “what are people's experiences of involvement in research?” Ethical approval was granted by the Berkshire Research Ethics Committee (ref: 12/SC/0495). We interviewed 38 people, using a maximum variation sampling approach,^27^ designed to capture the broadest possible range of different types of experience. Variation was sought across demographic characteristics (such as age, gender, ethnicity and socio‐economic status), and different types of involvement experience and length of involvement (see Table 1). Recruitment packs were distributed through a range of avenues, inviting people to contact us if interested in taking part. These avenues included NHS and university research networks and researchers, NIHR INVOLVE, social and print media advertising, medical charities, PPI coordinators and word of mouth.

**Table 1:** Self‐reported characteristics of interview participants (N=38)

Characteristic	Number of participants
Gender	
Male	20
Female	18
Ethnicity	
White British	35
White European	1
British Pakistani	1
Black British/Jamaican	1
Age	
18‐44 yr	5
45‐64 yr	17
65+ yr	16
PPI role^a^	
Patient	24
Carer	9
Dual patient and carer	1
Member of the public	4
Experience of involvement in research	
0‐5 yr	13
5‐10 yr	12
More than 10 yr	13

a Participants preferred many different role names, but for the purposes of this study, we have grouped them into these four categories.

Interviews were conducted in the individual's home (or elsewhere if preferred) and were video or audio recorded. The interview started with an unstructured section, in which people were invited to tell their story of involvement in health research. Semi‐structured prompting was used to explore specific areas, including how they became involved and why; how they defined the purpose of involvement; what tasks and activities they undertook; what helped or hindered involvement; their relationship with researchers; personal benefits and costs (financial and emotional) of involvement; and their reflections on a range of issues such as payment, diversity in involvement, impact of PPI and how involvement has changed over time. The development of these prompts was guided by the literature and the input of an advisory panel which included six patients, carers and members of the public (including two now working in research into PPI), as well as clinical and social science researchers. One of the patients on the panel is a co‐author of this study.

Interviews were transcribed verbatim; participants reviewed their transcripts and removed sections they did not wish to be used before assigning copyright to the University of Oxford for the data to be used for research, teaching, publication and dissemination online and through the creation of audio‐visual training and service improvement resources.

All data were coded and thematically analysed by LL and AMB. A modified grounded theory approach was employed, using constant comparison between each transcript and previously coded data to refine the coding framework and draw out both anticipated and emergent themes. This combination of inductive and deductive reasoning reflected the fact that we expected power relations, for example, to be a key factor in people's experiences, but we invited participants to talk about relationships with researchers in their own terms rather than imposing theoretical questions. The role of symbolic capital emerged as one of the key findings during the analytic process and is the focus of this study.

Lay summaries of the key themes across the whole data set (reviewed and approved by the expert advisory panel) are publicly available on the health website healthtalk.org. Pseudonyms are used below to preserve anonymity.

LL was the principal investigator and led the recruitment and analysis. Data collection was led by co‐author AMB. RS and SS were members of the advisory panel. All co‐authors were actively involved in analysis and theoretical development of the study, through meetings, iterative written exchanges and a half day of discussion and reflection to progress the analysis. All co‐authors have an interest in research on PPI, and one is a service‐user researcher who has lived with an incurable long‐term condition since childhood.

## 3 FINDINGS

Compared to other forms of capital, symbolic capital, the status and respect an individual commands in a particular context, is an under‐explored area and one which our findings suggest can contribute to understanding how changes in power may be negotiated. Below we examine the exercise of symbolic capital within four categories which emerged through our analysis: illness experience, technical illness knowledge, the challenging outsider and instability of symbolic capital. We then look at the extent to which a gradual shift in power is reported.

### 3.1 Illness experience

At one level, people described to us a status as an “ill person” similar to that of returning veterans. The military metaphor is not one we wish to pursue at length; while talk of “battling cancer” is common, it is also highly problematic even in theoretically curable physical conditions. In long‐term conditions and disability, there is of course no possibility of being a “returning veteran of illness” and metaphors of battling and heroically overcoming become even less appropriate.^28^ Nonetheless, similarities can be observed between the returning veteran—who has symbolic capital by virtue of having seen and experienced things most of us do not expect to, who is changed forever by the insights acquired—and the patient or service user. Many people we spoke to felt it was this general ability to help researchers catch a vivid glimpse of the foreign territory of illness and its real impact on life that gave them status and value, as much as any specific tasks such as rewriting a trial information leaflet or improving the design of a study.

Penny described this as “being the person who walks into the room who is terrified for their own or their child's health….You don't know what it's like until you've been that person at home, trying to eat a dinner and throwing up at the thought of the person opposite you dying.”

Julia said sometimes the raw emotion of her experience caring for her mother came through at meetings and she “broke down,” but how that was a good thing if it drew researchers’ attention to the reality of dementia, “not working with it, but actually living with it.”

In these cases, simply conveying some sense of the reality and emotions of lived experience of illness to researchers was the contribution people felt they brought—and as Julia indicates, this may come at some personal cost. Interestingly this may be an example where the logic of practice—not crying in meetings—is at odds with the expression of symbolic capital.

### 3.2 Technical illness knowledge

Alongside the currency of illness experience, symbolic capital might be based more on a specific kind of expertise derived from lived experience. Particularly in the case of long‐term conditions where self‐management is key, this may take the form of technical knowledge about treatment, or the nature of the condition, which might be considerably more in‐depth than the researcher's.

Helena recalled commenting on a research proposal about her condition and explaining to the researcher why it would be poor research. “I showed her why the questions weren't adequate, given this wealth of knowledge…I have about the condition…She needed to understand that what was standard belief about the condition is really crude.”

Here, symbolic capital might begin to merge into cultural capital, where the individual has the language and scientific understanding to meet the researcher in their cultural territory on more equal or even superior terms. But we suggest this is distinct from and more factually specific than the cultural capital described above where a person can draw on their education in another field to demonstrate they already have the same type of cultural capital as the researcher. It is a form of “earned capital” which legitimates people's symbolic capital derived from illness experience with an extra layer of power and respect through demonstration of detailed superior technical knowledge.

Helena described the interplay between illness credibility and technical skills thus “I have sat in meetings where people have used kind of academic games to slightly intimidate or shuffle for power. […] I can cut through all their games with something unanswerable, like, ‘well I live with this every day and I can't go home at the end of the research and forget about it, and what's important to me is….’ I can also offer knowledge that they just don't have, because the only things they know about my long term condition are the things they've learnt in textbooks, which cover about ten per cent of the things you actually need to know to live with it. So I can answer their practical questions in a way that is genuinely useful to them.”

At the same time, such knowledge may not be accepted as a form of capital if the researcher is unwilling to believe or listen to the individual, and their intervention is perceived as disruptive or out of place, as we explore in the next section.

### 3.3 The challenging outsider

Of course people who get involved in research may not have a relevant illness background, they may volunteer as a member of the public. A further form of symbolic capital, relevant to both patients and members of the public, could be based on being “the outsider” who can question taken‐for‐granted practices. People used terms such as “creative stupidity,” “fool” and “idiot,” suggesting they deliberately self‐identified with a stance of naivety, ignorance and lack of expert status, sometimes combined with an overt element of subversion—Geoff, for example, described himself as “a congenital anarchist.” People also described how professionals were sometimes relieved someone else had asked questions they themselves could not ask for fear of undermining their own status. (See Box 2)

Box 2 **The symbolic capital of the challenging outsider**

**Carla**

“Creative stupidity” I think is something that my partner calls it. It's sitting there and being bloody‐minded about making people spell things out in full, absolutely, and just pretend that I don't know anything. Which is as much for me, as it is for whoever it is who's doing the research that they want people to be engaged in. [I] just pretend I know nothing.

**Ceri**

I was actually in quite a privileged position because the other people round the table, what they said was being judged by their peers. And actually their professional reputation was on the line…whereas for me there was no consequence. It was just really I felt like I was a fool or not, which is no big deal…I can live with that. They were actually taking a much bigger risk in speaking at these panels.

**Colin**

We had our first meeting and I have to say that, quite frankly, we were both…gobsmacked because we didn't understand what was going on. Some of the words they used were quite intimidating… …It starts off with utter bewilderment: ‘what am I doing here? I haven't the foggiest idea! Dear me, I'm out of my depth’….Let me out of here’…And then eventually you reach the stage where you understand most of it and you feel, I call it the breakthrough moment when you think, ‘OK I know where I'm at, I know what I can do, I know my limitations, what can I do to push the project forward?’ And that's really when you start being productive.

**Interviewer**: What prevented you from running away?

**Participant**: Pride. I don't like giving up.

Geoff, in one part of his narrative, described himself first as “just an ordinary chap”—the common man who has little apparent capital in the face of the “eminently qualified.” Despite this initial self‐positioning as inexpert, he went on to counter this with his own expertise as a carer, and—crucially—the ability to see the “obvious, ordinary, everyday common sense answer” which researchers “wrapped up in the academic side of things…sometimes miss.” In doing so he challenges researchers’ apparent capital.

While some people adopted a semi‐jovial position of “asking the stupid question,” the outsider position could be presented in a more combative way. Penny was adamant that her outsider status did not diminish over time:

I'm not an expert and not only that, because I have been outside the system…I see it as a user. I don't think that they can ever see it as a user…So PPI representatives should never feel that they're on the same side and I don't think you do.

At the same time, the position of naivety was often not a route to symbolic capital at all, but rather an expression of genuine powerlessness. Colin (a healthy volunteer rather than a patient) described moving from bewilderment to acquiring a degree of cultural capital through learning about research, rather than deliberately using naivety (see Box 2). It is of note that, as a healthy PPI volunteer, Colin by definition had no illness experiences to draw on.

### 3.4 Instability of symbolic capital

Some people were concerned about a possible weakening of their symbolic status over time, and coming to conform too closely to researchers’ habitus. Alex explained “I don't think you ever stop feeling like a patient, but the intensity of it varies depending on what you're doing.” He went on to say that one needs to “continue to remind yourself of what it felt like.”

Despite having a long‐term condition, which arguably makes someone less likely to “forget” their patient experience, Carla was conscious of moving closer to a researcher view of the world, which might mean “you're no longer doing the job of defending the absolute lay person.”

This was one example of the unstable or contingent nature of symbolic capital, and how its currency may “wear thin.” Another concern was the danger of exclusion as a result of being perceived to threaten research habitus too directly. Bernard reflected on how those involved in the early days of PPI had faced this dilemma and had “acquiesced, perhaps, a little too much… because if you're troublesome, the doors can easily close.”

There were other instances where people recognized a need to play by certain rules to remain at the table. Penny described how “If you want to influence them [the researchers], it's best to be able to be rational and objective when commenting.” There was even a degree of closing ranks with researchers against the “difficult” PPI person (Box 3).

Box 3 **The instability of symbolic capital**

**Penny**

Because it isn't about only delivering your case to the table, otherwise it sounds like you're the one person beating the drum for your one condition…where what you're actually required to do is to be able to sit with a group of up to maybe eight or ten people who are experts in their field and to contribute—rationally. I believe if you want to influence them it's best to be able to be rational and objective when commenting.

**Ceri**

I know of some professional participants who do have an agenda that they take wherever they can get heard. That can be a problem…..Mostly, if people have a very clear idea of what would be really useful for them to do, they will cooperate with that and do it. I think the danger of the hobby horse riding is most apparent where there is not a clear guide to…what is wanted from people and what they can usefully contribute, and then they tend to fall back on what they always say.

Bernard, however, pointed out that being “troublesome” could be a *result* of exclusion as much as its cause “they'd only be troublesome because they couldn't go through the door.”

If symbolic capital overlaps into cultural capital, there can be a tension; they may work together to give the patient increased power, but if a patient's symbolism relies on a “naïve” role, learning about research habitus may cancel out symbolic capital. Helena, who went on to complete a PhD about her long‐term condition, described the complicated interplay between researchers and patients: “I've got researcher value and patient value at the same time. That can feel quite powerful. But it can also be really uncomfortable. If you want to use influence to change something, which hat do you pull out”? Once she had completed her doctorate, she found that she was formally excluded from taking part in some institutions’ PPI because those with PhDs were not accepted as “patients.”

Symbolic capital of this kind does not have permanent currency and only exists if it is recognized and valued by a different and more powerful group (in this case researchers). It is not extended automatically but rather granted in specific circumstances. This value may be eroded if those with power consider that patients’ “creative stupidity” is overstepping the mark, is too challenging of the status quo or otherwise inconsistent with researchers’ assumptions about patients.

### 3.5 Gradual Shift in Power

Despite the instability of symbolic capital at an individual level, the comment that involvement “might just change the equilibrium” reflected a discourse in the interviews that a collective shift in power was occurring over time. As one participant said, “Take on board that the paradigm's shifted, it's changed. Citizen researchers are now going to be part of academic life.” This change was attributed to both the prioritization of involvement by funders and other leaders, such as the Chief Medical Officer; a change in research culture, with younger researchers perceived as more keen to involve; but also to the significant efforts of lay people.

In Norma's experience (Box 4), the relationship between researchers and patients has matured over the years. She described how researchers used to infantilize patients by not critically reviewing their ideas or contributions. But this has changed “partly down to more experience, more confidence and competence on the part of the people doing the involvement.”

Box 4 **Gradual shift in power**

**Norma**

Something that used to irritate me was…a patient would say something in a meeting, and somebody would go, “Oh that's fantastic, thank you so much”. And it wouldn't be fantastic… It's like the inverse of this:…when people actually feel…able to disagree with you or challenge you back on something…it's grown up enough to have that kind of discussion. And so if that's the grown‐up end of the relationship between us and the researchers, then the other one I was attempting to describe is like a more infantile version of…patting you on the head, feeling that you've got to…welcome everything. Well actually you should look at our ideas as critically as we look at yours…I see that less now. And that possibly is partly down to more experience, more confidence and competence on the part of people doing the involvement. Partly that's because more people are trained. Younger generations of researchers coming through seem to feel more open to the benefits, but people who made their careers made their reputations 20/30 years ago possibly never had to get involved with us as patients in that kind of relationship when they were building their careers…

Reflecting on his past experiences, Philip said there was a danger of tokenism and that “you were there for the sake of the paperwork.” A gradual shift has led to “research projects where they've got co‐applicants…and partnership right from the start.”

Elizabeth felt that people could now safely take more of a stand without researchers seeing them as “somebody who's going to hold up their study or put a spanner in the works.”

People acknowledged that the changes they perceive do not mean that involvement is universally accepted or valued and that some researchers may view it as a “tick‐box” exercise. However, it was suggested that the degree of funder endorsement of involvement was gradually constraining researchers’ own power to resist it.

There's still dinosaurs out there…But among the ones who have come out of the dark ages then yes it's very much accepted because a lot of them won't progress or won't proceed with their proposals or their studies or whatever without contacting consumers.

[Bill]

## 4 DISCUSSION

Problems of unequal power have consistently been documented in studies of public participation; our findings are unsurprisingly consistent with this literature but add to our understanding of how different forms of capital operate. We have shown how the illness experience and technical illness knowledge are forms of symbolic capital, and how patients and public can position themselves as challenging the norms of current academic practice. We question the stability of their symbolic capital, but note a gradual shift in power to which involvement has contributed. Specifically this study is the first to consider conceptually how different forms of symbolic capital operate and their critical role in challenging the balance of power between researchers and those involved in PPI.

It is of course open to question how real any perceived exercise of power is. Those who hold most of the cards—in this case the research community—still have the power to decide whom they will involve, and how far and in what ways they will cede elements of power to the people they invite in, or rather co‐opt them to their way of thinking. It has been argued, for instance, patients’ perspective may be “‘tamed’ to make it more congruous with that of the professional researcher,”^29^ although this has been disputed.^30^

One problem with this discourse is that it risks characterizing patients in PPI as lacking agency and ultimately always under the thumb of researchers. The example of other social movements, for example in disability and women's health, suggests people can exercise collective agency over time to bring about a broader shift in attitudes to professional power and knowledge. Our sense from the data is that “tamed” is too passive a word to represent the knowing, reflective and critical ways in which the people in our sample made sense of their PPI experiences, and how they sought to negotiate influence. Whilst acknowledging the possibility of losing their “patient‐ness” and sharing their enthusiasm for and enjoyment of research, many were acutely aware of continuing power inequalities, articulate about the strategies they adopted and alert to the need to “play the game.” Our findings suggest that while there may be a continuing “shaping of desires” by researchers, as Lukes^19^ would describe it, and suppression of certain topics, people could and did find ways to question and challenge the status quo. We have suggested several distinctive ways in which symbolic capital may be actively wielded to redress the power imbalance.

Symbolic capital is of particular interest as it does not necessarily depend on other forms of capital and may even be seen as countercultural. The conscious and challenging self‐positioning as “fool,” “anarchist” and exponent of “creative stupidity” has some resonance with the figures of the Lord of Misrule or the court jester, who are given permission to overturn normal custom and practice, to say the unsayable, or “speak truth to power.” In the context of PPI, this can be the deliberate overturning of the research habitus and hierarchy in favour of a different form of expertise and the bringing of emotion into the normally controlled and “objective” research space. However, symbolic capital may be more unstable than other forms of capital, and more dependent on those with greater power recognizing it.

Dowding^31^ argues that those who benefit from power may not realize they are doing so. He suggests that “once we realise how our institutions affect the interests of ourselves and others, then anyone who does not act to change those institutions for the better is part of the structure of domination” (p. 142). We acknowledge that researchers may themselves feel powerless sometimes, whether because of university hierarchies, pressure from funders or ethics committees, or existence on short‐term contracts. Inevitably casting researchers as one (powerful) group oversimplifies divisions and power relationships within the research community. Easy assumptions about powerful and non‐powerful patient advisers also oversimplify the picture. This is consistent with Bourdieu's^21^ argument that differing forms of capital may be variably distributed both within and between different classes.

## 5 CONCLUSION

Our findings represent a challenge for all sections of the health research community to reflect on the capital and power it wields, but also to acknowledge the ways in which patient involvement is challenging the research status quo. The tools and processes of PPI are underpinned by tacit assumptions and models of capital which are shifting. Callaghan and Wistow,^18^ in their study of forms of capital in patient participation in healthcare services, suggest that communities which may not have much cultural capital “may possess other forms of capital, which provide a basis for dialogue with professionals. Questioning the dominant understanding lays bare the exercise of power by professionals, something previously represented largely in terms of expert knowledge and competence” (p. 597). They suggest that health is a changing habitus, partly driven by “the undermining of professional cultural capital's claims and the associated reduction in [doctors’] symbolic capital’ but also an increased value ‘placed on the form of knowledge and rights to speak held by patients and public.”

Patients who are participating in these changes may also find it helpful to reflect on their relationships with researchers in terms of symbolic capital. For example, if the rhetoric of a PPI group focuses on the importance of the naïve patient, those wishing to have more influence in that group must either directly challenge that symbolism or learn to frame their involvement in terms the researchers’ value: the “everyman” or the outsider who needs to ask questions. In such a group, showing familiarity with research methods may actually erode a patient's influence. Patients who are aware of their symbolic capital and the hidden assumptions shoring it up may be less vulnerable to actions by health researchers that devalue or discredit their contribution. Patients who are able to invoke other forms of capital—such as their tertiary education or their familiarity with committee behaviour—may be able to use this to bolster their symbolic capital and retain status in the group in the face of otherwise devaluing behaviour, but for others, such behaviour may effectively deprive them of all status.

This study is limited to a qualitative UK‐only sample. Although the demographic profile of the sample was mixed, it was easier to recruit people with higher levels of education, of white British origin. This is not only a common problem in health research generally, but also reflects the typical profile of people involved in PPI. People from less advantaged groups may find it harder to exercise symbolic capital. While this study focused on PPI in a UK context, key concepts may be transferable to other country settings. We would urge others involved in PPI to consider how different forms of capital operate in their country contexts and how they could utilize this knowledge to enhance the potential impact PPI can make on research and on the people involved in research.

## CONFLICT OF INTEREST

The authors declare no conflict of interest.

## END NOTE

1 There are many debates about terms used to describe people who get involved in PPI. We use the word “adviser” in preference to the common term “representative,” on the grounds that it is misleading and unrealistic to expect “representativeness.”

## References

[1] Coulter A . Engaging Patients in Healthcare. Maidenhead: Open University Press; 2011.

[2] Berwick DM . What ‘patient‐centered’ should mean: confessions of an extremist. Health Aff. 2009;28:w555–w565. 10.1377/hlthaff.28.4.w555 19454528

[3] Crawford MJ , Rutter D , Manley C , et al. Systematic review of involving patients in the planning and development of health care. BMJ. 2002;325:1263. 12458240 10.1136/bmj.325.7375.1263 PMC136920

[4] Bate P , Robert G . Experience based design: from redesigning the system around the patient to co‐designing services with the patient. Qual Saf Health Care. 2006;15:307–310. 17074863 10.1136/qshc.2005.016527 PMC2565809

[5] Wiig S , Storm M , Aase K , et al. Investigating the use of patient involvement and patient experience in quality improvement in Norway: rhetoric or reality? BMC Health Serv Res. 2013;13:206. 23742265 10.1186/1472-6963-13-206 PMC3680039

[6] Thornton S . Beyond rhetoric: we need a strategy for patient involvement in the health service. Br Med J. 2014;348:g4072. 24957899 10.1136/bmj.g4072

[7] Coulter A , Locock L , Ziebland S , Calabrese J . Collecting data on patient experience is not enough: they must be used to improve care. Br Med J. 2014;348:g2225. 24671966 10.1136/bmj.g2225

[8] Beresford P . User involvement in research and evaluation: liberation or regulation? Soc Policy Soc. 2002;1:95–105.

[9] Snape D , Kirkham J , Britten N , et al. Exploring perceived barriers, drivers, impacts and the need for evaluation of public involvement in health and social care research: a modified Delphi study. BMJ Open. 2014;4:e004943. 10.1136/bmjopen-2014-004943 PMC4067891 24939808

[10] NIHR INVOLVE . What is public involvement in research? http://www.invo.org.uk/find-out-more/what-is-public-involvement-in-research-2/. Accessed January 29, 2016.

[11] NIHR INVOLVE . Briefing note three: why involve members of the public in research. http://www.invo.org.uk/posttyperesource/why-should-members-of-the-public-be-involved-in-research/. Accessed January 29, 2016.

[12] Brett J , Staniszewska S , Mockford C , et al. Mapping the impact of patient and public involvement on health and social care research: a systematic review. Health Expect. 2014;17:637–650. 22809132 10.1111/j.1369-7625.2012.00795.x PMC5060910

[13] Gibson A , Britten N , Lynch J . Theoretical directions for an emancipatory concept of patient and public involvement. Health (London). 2012;16:531–547. 22535648 10.1177/1363459312438563

[14] NIHR. Going the extra mile: Improving the nation's health and wellbeing through public involvement in research. http://www.nihr.ac.uk/documents/about-NIHR/NIHR-Publications/Extra%20Mile2.pdf. Accessed January 29, 2016.

[15] Gibson A , Lewando‐Hundt G , Blaxter L . Weak and strong publics: drawing on Nancy Fraser to explore parental participation in neonatal networks. Health Expect. 2011;17:104–115. 22040481 10.1111/j.1369-7625.2011.00735.x PMC5060698

[16] Fraser N . Rethinking the public sphere: a contribution to the critique of actually existing democracy In: Fraser N, ed. Justice Interruptus: Critical Reflections on the ‘Postsocialist’ Condition. London: Routledge; 1997:109–142.

[17] Hutchison K , Rogers W , Entwistle VA . Addressing deficits and injustices: the potential epistemic contributions of patients to research. Health Care Anal. 2016. doi:10.1007/s10728‐016‐0323‐5. 10.1007/s10728-016-0323-5 27277736

[18] Callaghan GD , Wistow G . Publics, patients, citizens, consumers? Power and decision‐making in primary health care. Public Adm. 2006;84:583–601.

[19] Lukes S . Power: A Radical View. Basingstoke: Palgrave Macmillan; 2005.

[20] Webb J , Schirato T , Danaher G . Understanding Bourdieu. London: Sage; 2002.

[21] Bourdieu P . What makes a social class? On the theoretical and practical existence of groups. Berkeley J Sociol. 1987;32:1–17.

[22] Beresford P . Beyond the Usual Suspects. London: Shaping Our Lives; 2013.

[23] Petit‐Zeman S , Locock L . Health care: bring on the evidence. Nature. 2013;501:160–161. 24032134 10.1038/501160a

[24] Rickard W , Purtell R . Finding a way to pay in the UK: methods and mechanisms for paying service users involved in research. Disabil Soc. 2011;26:33–48.

[25] Bourdieu P . The Logic of Practice. Cambridge: Polity Press; 1990.

[26] MacLean A , Kleykamp M . Coming home: attitudes toward U.S. veterans returning from Iraq. Soc Probl. 2014;61:131–154.

[27] Patton MQ . Qualitative Evaluation and Research Methods. Newbury Park, CA: Sage; 1990.

[28] Sontag S . Illness as Metaphor. New York: Farrar, Strauss and Giroux; 1978.

[29] Ives J , Damery S , Redwood S . PPI, paradoxes and Plato: who's sailing the ship? J Med Ethics. 2012;39:181–185. 22267385 10.1136/medethics-2011-100150

[30] Staley K . There is no paradox with PPI in research. J Med Ethics. 2013;39:186–187. 23288267 10.1136/medethics-2012-100512

[31] Dowding K . Three‐dimensional power: a discussion of Steven Lukes’ ‘power: a radical view’. Polit Stud Rev. 2006;4:136–145.

